# Is the answer to clinical questions provided by Bayesian network meta-analysis? Regarding the optimal duration of DAPT and the best subsequent SAPT

**DOI:** 10.1093/ehjcvp/pvaa032

**Published:** 2020-05-19

**Authors:** Shinichi Goto, Shinya Goto

**Affiliations:** 1 Department of Cardiology, Keio University School of Medicine, Japan; 2 Department of Medicine (Cardiology), Tokai University School of Medicine, Japan

**This editorial refers to ‘De-escalation of antiplatelets after percutaneous coronary intervention: a Bayesian network meta-analysis of various de-escalation strategies’, by S.U. Khan *et al*., pp. 209--215.**

Antiplatelet therapy is strongly recommended in patients who have undergone percutaneous coronary intervention (PCI) and stenting. PCI in nature causes endothelial damage and subsequent platelet accumulation.^1^ Historical clinical trials clearly demonstrated a marked decrease in stent thrombosis by dual antiplatelet therapy (DAPT) with aspirin and a P2Y_12_ inhibitor.^2^ DAPT is the established standard of care in patients treated with PCI, especially with a stent, at least for 1 month.^3^

Despite several reports demonstrating the lower cardiovascular (CV) events in patients treated with extended DAPT (12 months or longer),^4^^,^^5^ shorter DAPT is favoured now due to increased bleeding risk with longer DAPT. Indeed, many recent trials suggested non-inferior clinical outcomes with shorter DAPT compared with standard 12-month duration.^6^ The best time to switch from DAPT to a single platelet agent (SAPT), and which antiplatelet agent is best for long-term SAPT, is still unknown. It is not efficient to conduct various randomized clinical trials to find the best duration of DAPT and the best subsequent SAPT without any clues.

In this issue of the *European Heart Journal* – *Cardiovascular Pharmacotherapy*, Khan *et al*. have presented the results of a Bayesian network meta-analysis on seven randomized controlled trials (RCTs) comparing 12-month DAPT vs. 1-month DAPT followed by a P2Y_12_ inhibitor, 3-month DAPT followed by a P2Y_12_ inhibitor, and 3-month DAPT followed by aspirin.^7^ Typically, meta-analyses are conducted to provide more robust clinical evidence by accumulation of replicated two-way comparisons.^8^ Bayesian network meta-analysis is unique in that it enables three-way or more comparisons. Using the same term ‘meta-analysis’, the meaning of the results from a ‘Bayesian network meta-analyses’ differ substantially from those of a traditional ‘meta-analysis of pairwise comparison’.^9^ Here, the authors are attempting to provide the answer to a three-way comparison for two clinical questions. It is a nice idea to apply Bayesian network meta-analysis for clinical studies to find the current best answer for the best duration of DAPT and best candidate for long-term SAPT. Six out of the seven trials Khan *et al*. included in this network meta-analysis showed only non-inferiority of shorter DAPT as compared with 12-month DAPT with regard to bleeding.^10–15^ With the Bayesian network meta-analysis, Khan *et al*. showed statistically fewer all bleeding events in the group of patients treated by 1- or 3-month DAPT followed by a P2Y_12_ inhibitor [hazard ratio (HR) 0.28, 95% confidence interval (CI) 0.10–0.81, and HR 0.57, 95% CI 0.33–0.98, respectively as compared with 12 month DAPT]. On the other hand, the 3-month DAPT followed by aspirin did not reduce the risk of bleeding (HR 0.75, 95% CI 0.45–1.25 as compared with 12-month DAPT). Of particular note, there were no trends of increased risk of myocardial infarction or stent thrombosis with 1- and 3-month DAPT followed by a longer duration of a P2Y_12_ inhibitor as compared with 12-month DAPT in the analysis provided by Khan *et al*. The results suggested the shorter duration of 1- or 3-month DAPT followed by a P2Y_12_ inhibitor to be the best antiplatelet therapy in patients after PCI.

Bayesian network meta-analysis is a reasonable tool to analyse the accumulated clinical data. However, there are obvious limitations in regard to application of the obtained results for clinical practice. First, only accumulated data could be analysed by the Bayesian network meta-analysis. If the ideal duration of DAPT was the period never tested in clinical trials such as 1.5 months or 2 months, the ‘ideal’ duration of DAPT could not even be determined by Bayesian network meta-analysis. Secondly, there is a huge selection bias from 6203 screened trials to only 7 trials actually analysed. There is selection bias from real-world clinical practice to clinical trials. Bayesian network meta-analyses even amplify the potential selection bias. Thirdly, even within seven trials, patient recruitment time differed substantially. Clinical outcome with standard of care improved over time. The higher clinical event rates in earlier trials may influence the overall results. Indeed, two of the three trials that allocated aspirin for SAPT were published earlier (RESET and OPTIMIZE trials were published in 2012^16^ and 2013^17^) despite all the trials that allocated a P2Y_12_ inhibitor as SAPT being published after 2018. Caution is needed to recommend a P2Y_12_ inhibitor in clinical practice because fewer bleeding events in that arm may only reflect the general improvement of clinical outcome over time. We still need a head-to-head comparison of aspirin with P2Y_12_ inhibitors after short-term DAPT in PCI patients.

In conclusion, the authors are commended for publishing their results utilizing Bayesian network meta-analysis. Lower bleeding with 1- or 3-month DAPT followed by SAPT as compared with 12-month DAPT is confirmed. A better outcome with a P2Y_12_ inhibitor than aspirin is suggested. The authors provide good clues to select which clinical questions should be solved by future head-to-head comparison.

**Conflict of interest:** Shinichi Goto has nothing to disclose. Shinya Goto received research grants from Sanofi, Pfizer, and Ono, independent research grant support from Bristol-Myers Squibb (33999603), and personal fees from the Thrombosis Research Institute (London, UK) as a member of the Steering Committee for GARFIELD-AF and GARFIELD-VTE, and from the American Heart Association as an Associate Editor.

## Notes

The opinions expressed in this article are not necessarily those of the Editors of the *European Heart Journal* – *Cardiovascular Pharmacotherapy* or of the European Society of Cardiology.
